# Primary Intracranial Sarcomatoid Yolk Sac Tumor With Unique Histology: A Case Report

**DOI:** 10.1155/crip/2011129

**Published:** 2025-12-07

**Authors:** Jessica A. Ortega-Balderas, Hayde S. Ramos-Marrero, Raquel Garza-Guajardo, Oralia Barboza-Quintana, Barbara Saenz-Ibarra

**Affiliations:** ^1^ Department of Anatomical Pathology and Cytopathology, University Hospital “Dr. José Eleuterio González”, Monterrey, Nuevo Leon, Mexico

**Keywords:** case report, intracranial tumor, midline tumor, pure germ cell tumor, yolk sac tumor

## Abstract

Primary intracranial pure yolk sac tumors (YSTs) are rare, and their typical histological findings have been documented in the literature. We report a case of a 20‐year‐old young man with an intra‐axial primary YST exhibiting a unique sarcomatoid morphology. This case demonstrates that primary intracranial YSTs present uncommon patterns underscoring the importance of including YSTs in the differential diagnosis of midline masses. To the best of our knowledge, this is the first reported pure primary intracranial YST showing sarcomatoid histology.

## 1. Introduction

According to the fifth edition of the WHO Classification of Tumors of the Central Nervous System (CNS), yolk sac tumors (YSTs) are composed of primitive‐looking cells embedded within variable cellular and myxoid stroma, but there are many recognized patterns (WHO Classification of Tumours Editorial Board. 47 World Health Organization Classification of Tumours of the Central Nervous System. 5th 48 Ed.Lyon: [1]). It is diagnostically useful when there are hyaline globules and Schiller‐Duval bodies, both positive for alpha‐fetoprotein (AFP), and when the epithelial cells are positive for Sal‐like protein 4 (SALL4), glypican‐3 (GPC‐3), and cytokeratins [1, 2]. Although not described in the CNS Germ cell tumours (GCT) section, the sarcomatoid pattern is a recognized histological variant of YSTs. This pattern is described by the WHO 5th Edition as a progression of the myxoid pattern, where the spindled cells are dispersed in the stroma with myxomatous and microcystic components.

Primary pure YSTs are rare, as they are often a component of mixed GCTs. The extragonadal primary YSTs most commonly arise in the mediastinum, retroperitoneum, and CNS, and the symptoms vary according to their localization (most commonly in pineal and sellar regions) and include hypopituitarism, diabetes mellitus, visual acuity problems, seizures, and hydrocephalus [1, 2]. There are no standard therapeutic options; however, neoadjuvant therapy may shrink tumor size and aid resection [2]. Despite its poor prognosis, total resection followed up with adjuvant chemotherapy is recommended to improve the patient′s outcome [3].

There is an increasing number of case reports, describing unusual locations, multifocality, and variable clinical presentations. We present a 20‐year‐old man, with no remarkable clinical history, who was diagnosedwith a primary intracranial YST exhibiting a unique sarcomatoid morphology.

## 2. Case Report

A 20‐year‐old man, with no remarkable medical history, was admitted to our institution with blurry vision, headache, nausea, and vomiting. On examination, he showed Parinaud syndrome, a left Babinski sign, left hypoesthesia, and left hemiparesis. Computed tomography (CT) revealed a solitary intra‐axial lesion measuring 5.1 × 4.7 cm, involving and compressing the thalami, mesencephalic tegmentum, cerebral peduncles, and pineal region. Additional imaging ruled out other tumors.

### 2.1. Pathological Findings

**Figure 1:**
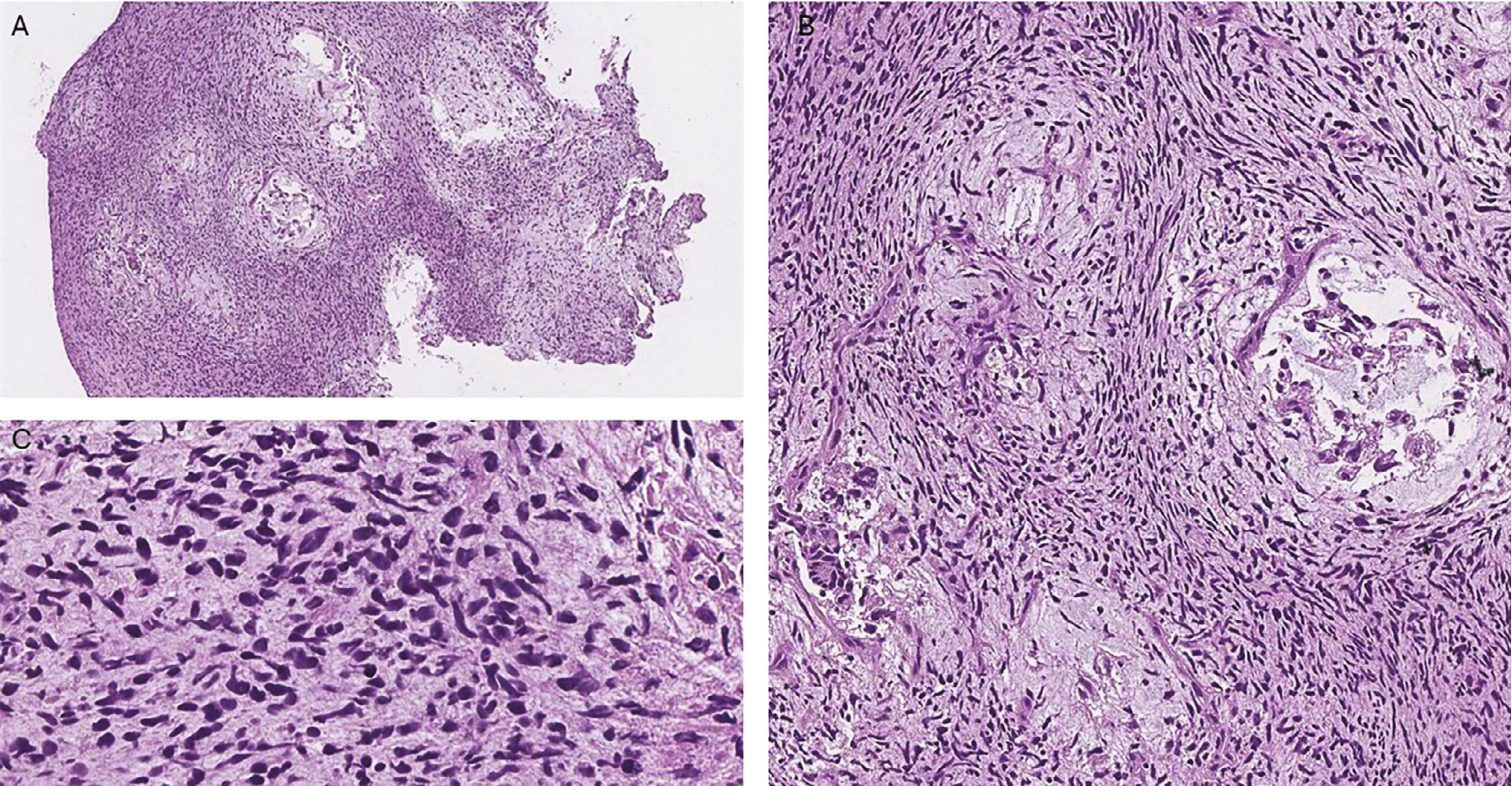
(A) Frozen section (H&E, 10×) showing nests of epithelioid pleomorphic cells within myxoid pools surrounded by a fibrillary stroma with small spindled basophilic cells. (B) Frozen section (H&E 40×) showing the sarcomatoid basophilic cells within a fibrillary stroma. (C) Definitive specimen (H&E, 40×) showing spindled basophilic cells in a fibrillary stroma.

The initial frozen section of the biopsy showed nests of epithelioid cells in a microcystic pattern, embedded in a myxoid material (Figure 1A,B) along with a fibrillary stroma containing sarcomatoid basophilic spindled cells as shown in Figure 1C. Initially, this case was morphologically consistent with a high‐grade glial lesion, and preoperative serum AFP levels were not available at the moment of the first evaluation.

**Figure 2:**
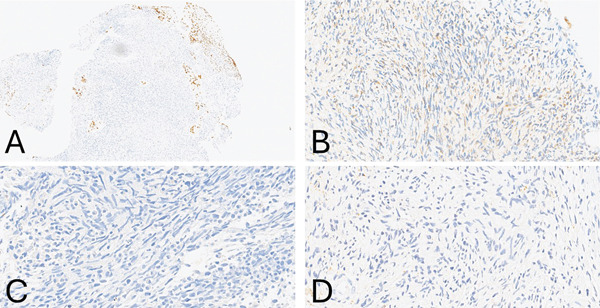
(A) Immunohistochemistry markers were positive for SALL4 in the epithelioid pleomorphic cells and for (B) glypican‐3 in the spindled cell component. The tumor was negative for (C) GFAP and (D) S100 markers.

The definitive histological examination with additional resected tissue revealed more spindled cells within a fibrillary and myxoid stroma. The initial immunohistochemistry (IHC) markers′ panel of GFAP, S100 (shown in Figure 2C,D), and OCT3/4 were negative. During the biopsy workup and upon learning the patient′s serum AFP levels were 608.3 ng/mL, we performed SALL4, GPC‐3 (shown in Figure 2A,B), which were positive as shown, confirming the diagnosis of a pure primary YST with sarcomatoid features.

## 3. Discussion

We present a young man with a primary intra‐axial YST, with no other neoplastic lesions, characterized by spindled cells in a microcystic arrangement in a myxoid background, elevated AFP serum levels (unavailable at the initial workup), and positive germ cell IHC markers. However, due to the urgent and deteriorating clinical status of the patient, our assessment proceeded without knowing the tumoral marker levels.

Primary intracranial GCTs are exceedingly rare, and the distinction among each component is key as their treatment and prognosis are different. This tumor often presents in young patients without significant comorbidities [4], and the diagnosis requires MRI findings, along with laboratory and pathological results. Elevation of AFP serum levels is commonly seen in tumors containing yolk sac elements or teratomatous glands [5]. Thus, we provide an extra differential diagnosis for intracranial midline masses that are negative for glial and mesenchymal markers without the classical mixed GCT but with a challenging and unusual histology.

Our main differential morphological diagnoses included CIC‐rearranged sarcoma, primary intracranial sarcoma (DICER1‐mutant), diffuse midline glioma, diffuse pediatric‐type high‐grade glioma, sarcomas, and plasmacytic‐rich meningioma, as the sarcomatoid morphology suggested a glial origin. However, with the limited material, we could not perform as many IHC markers as optimal. Although neuroimaging is the initial diagnostic modality, the findings are not specific or pathognomonic for distinguishing them from astrocytoma or ependymoma, and misdiagnosis is possible [3, 4, 6].

Although there are morphologically similar cases, these include yolk sac elements that are a GCT component. García‐Muñiz et al. [7] described a similar sarcomatoid component. However, their IHC panel supported the diagnosis of a mixed GCT (with immature teratoma and YST). Mufti and Jamal also presented a reticular YST pattern [5]. Another case report described sarcomatous transformation in an intracranial teratoma of a 13‐year‐old girl [8]. In this context, complementary molecular analyses, such as sequencing for DICER1 mutations or CIC gene rearrangements can enhance diagnostic accuracy. For example, distinguishing this entity from gliomas or other intra‐axial tumors with similar histologic appearance (including the immature morphology of the DICER1 mutant sarcoma with chondroid differentiation), can be difficult. Additionally, weak and focal SALL4 and myogenin expression which could be misdiagnosed as a GCT [9]. Although such molecular testing was not feasible in the current case, these approaches are critical in future evaluations of sarcomatoid intracranial lesions. This use would strengthen diagnostic accuracy and better guide therapeutic planning.

Raynald et al. suggest a combination therapy with neoadjuvant chemotherapy and radiotherapy should be highly considered. However, they describe tumors with classical morphology, which naturally tells these tumors apart from other malignant GCTs. The standard of care and the efficacy of surgical resection combined with platinum‐based chemotherapy in the treatment of intracranial GCTs are well established [10]. But reviews by Corazzelli et al. [2] and Raynald et al. [3] highlighted there is not enough evidence on prognosis for pure YST tumors. Yet, pure YSTs are associated with poor prognosis and high mortality, given the complex anatomical structures involved and the difficulty of achieving total resection. Conversely, mixed GCTs located in the suprasellar and basal ganglia may have a favorable prognosis, regardless of histological type [5]. Future neurosurgical techniques will make it possible to improve survival rates [3]. The precise molecular differentiation plays a crucial therapeutic role, as DICER1 intracranial tumors′ treatment of choice is peripheral sarcomas adapted chemotherapy after surgery [11].

This case emphasizes the diagnostic challenges of primary intracranial YSTs with exclusively sarcomatoid morphology, particularly when limited available tissue, pathology testing, and urgent clinical circumstances restrict the use of complementary IHC and molecular studies. While serum AFP levels and GCT markers were supportive, molecular testing (DICER1 mutation or CIC rearrangement analysis) could have refined diagnostic precision for morphologically similar tumors. Given the rarity and poor prognosis of this YST, the awareness of this pattern, which could be easily misinterpreted as other midline intra‐axial neoplasms in young patients, will help ensure and avoid misdiagnosis, warrant appropriate addition of molecular tools, and optimize patient outcomes.

## Ethics Statement

The study was done in adherence to the institutional ethics committee guidelines (Hospital Universitario “Dr. José Eleuterio González”). As the study is based on tissue samples and no clinical trial was done nor registered in the ethics committee as per institutional ethics committee guidelines, the Institutional Review Board (IRB) number was waived. The study was performed in accordance with the principles of the Declaration of Helsinki.

## Consent

Written informed consents were obtained prior to the patient′s hospitalization, as per the hospital guidelines. The present work always makes the effort to uphold patient confidentiality.

## Conflicts of Interest

The authors declare no conflicts of interest.

## Author Contributions

Jessica A. Ortega‐Balderas and Hayde S. Ramos‐Marrero contributed equally to this work.

## Funding

No funding was received for this manuscript.

## Supporting information


**Supporting Information** Additional supporting information can be found online in the Supporting Information section. This case report was prepared in accordance with the CARE Checklist to ensure completeness of this work. The completed checklist is provided as supporting information.

## Data Availability

The data that support the findings of this study are available from the corresponding author upon reasonable request.
